# Implication of human endogenous retrovirus W family envelope in hepatocellular carcinoma promotes MEK/ERK-mediated metastatic invasiveness and doxorubicin resistance

**DOI:** 10.1038/s41420-021-00562-5

**Published:** 2021-07-08

**Authors:** Yan Zhou, Lijuan Liu, Youyi Liu, Ping Zhou, Qiujin Yan, Honglian Yu, Xiaobei Chen, Fan Zhu

**Affiliations:** 1 State Key Laboratory of Virology and Hubei Province Key Laboratory of Allergy & Immunology, Department of Medical Microbiology, School of Medicine, Wuhan University, 430071 Wuhan, P. R. China; 2 Wuxi School of Medicine, Jiangnan University, 214000 Wuxi, P. R. China; 3 Department of Biochemistry and Collaborative Innovation Center, Jining Medical University, 272067 Jining, P. R. China; 4 Department of Infectious Diseases, Renmin Hospital, Wuhan University, 430071 Wuhan, P. R. China

**Keywords:** Prognostic markers, Prognostic markers

## Abstract

Human endogenous retrovirus (HERVs), originating from exogenous retroviral infections of germ cells millions of years ago, have the potential for human diseases. Syncytin-1, an envelope protein encoded by the HERV W family, participates in the contexts of schizophrenia, multiple sclerosis, diabetes, and several types of cancers. Nevertheless, there is no report on the expression pattern and potential mechanism of Syncytin-1 in HCC. Here we found Syncytin-1 expression was up-regulated in HCC compared to adjacent non-tumorous tissues, especially in advanced HCC. Syncytin-1 was an independent risk factor to predict vascular invasion, metastasis, larger tumor size, and poor prognosis in HCC patients. Further analysis discovered that Syncytin-1 overexpression positively associated with HCC patients with serum HBsAg positive. Functional experiments in vitro and in vivo demonstrated that Syncytin-1 enhanced cell proliferation, metastasis, and tumorigenicity in HCC. Kyoto Encyclopedia of Genes and Genomes (KEGG) pathway analysis suggested that the mitogen-activated protein kinase (MEK)/extracellular signal-regulated protein kinase (ERK) pathway was involved in HCC. Our clinical data indicated that the levels of phosphorylation MEK1/2 and ERK1/2 were increased in HCC comparing with adjacent non-tumorous tissues. It showed the linear correlation between Syncytin-1 expression and upregulated MEK1/2 and ERK1/2 phosphorylation levels in HCC. Furthermore, Syncytin-1 activated MEK/ERK pathway in HCC cells. In-depth research showed that the inflammation-activated MEK/ERK pathway was essential in Syncytin-1 promoted hepatocarcinogenesis. Syncytin-1 suppressed doxorubicin-induced apoptosis via MEK/ERK cascade. In conclusion, Syncytin-1 promoted HCC progression and doxorubicin resistance via the inflammation-activated MEK/ERK pathway. Our findings revealed that Syncytin-1 was a potential prognostic biomarker and therapeutic target for HCC.

## Introduction

Human endogenous retrovirus (HERVs), which originate from exogenous retroviral infections of germ cells millions of years ago, are transmitted to the next generation in a Mendelian manner along with the human genome [1]. The HERVs elements comprise up to around 8% of the human genome [2, 3]. Only a small proportion of the elements encode proteins participating in various biological processes [4]. HERVs have been identified at least 55 families which are categorized into three main classes: Class I, Class II, and Class III [5]. HERV W family belonged to Class I, as a putative causative agent for multiple sclerosis (MS), is one of the most important members of the HERV family [6–8].

Syncytin-1, also known as ERVWE1, is a functional envelope glycoprotein encoded by a single HERV-W env locus that harbors a complete open-reading frame [9]. Syncytin-1 exerts critical functions in the placental trophoblastic formation and maternal immunosuppressive [10, 11]. Abnormal expression of Syncytin-1 participates in inflammation abnormalities of schizophrenia and MS [5, 12]. Recent studies have shown that overexpression of Syncytin-1 is implicated with several types of cancers, including endometrial cancer, breast cancer, leukemia, and urothelial cell carcinoma [13–16]. To the best of our knowledge, there is still no report underlying the role of Syncytin-1 in human hepatocellular carcinoma (HCC).

HCC is the fifth most common cancer and the second cause of cancer-related death worldwide, with more than 840,000 new cases in 2018 [17]. The 5-year survival rate of patients with HCC is 18%, which reflects that most patients failed to be diagnosed at an early stage [18]. Chronic hepatitis B or C virus (HBV, HCV) infections are the major causes leading to HCC [19, 20]. Our previous work has reported that HBV X protein (HBx) induced the overexpression of Syncytin-1 in HepG2 cell line via activating inflammation pathway [21], suggesting that Syncytin-1 might role as a cofactor in HCC pathogenesis. Several inflammation-activated pathways, including mitogen-activated protein kinase (MEK)/extracellular signal-regulated protein kinase (ERK) pathway, are induced in the development of HCC [22, 23]. Combined with Syncytin-1 are a potential oncogene in various metastasis tumors and its capacity to induce inflammation, it will be interesting to investigate the expression pattern of Syncytin-1 in HCC, as well as the relationship between Syncytin-1 and MEK/ERK signal in HCC.

In the present study, bioinformatics analysis showed that Syncytin-1 was highly expressed in HCC tissues compared to the corresponding non-cancerous liver tissues. Analysis in silico also pointed out that MEK/ERK pathway was involved in HCC. Our clinical data indicated similar results. Furthermore, overexpression of Syncytin-1 predicted higher tumor stages in HCC. Syncytin-1 was an independent risk factor to predict vascular invasion and poor prognosis in HCC patients. In-depth analysis of clinical data indicated that the levels of phosphorylation MEK1/2 and ERK1/2 showed a linear regression with the expression of Syncytin-1. In vitro and in vivo assay investigated that Syncytin-1 enhanced cell proliferation, metastasis, and tumorigenicity in HCC. Syncytin-1 was also involved in doxorubicin-resistance in HCC cells. The further study discovered that Syncytin-1 promoted hepatocarcinogenesis and drug resistance via MEK/ERK pathway. In conclusion, these results might provide novel insights into the mechanism underlying the development of HCC, as well as put forward potential therapeutic strategies of HCC.

## Results

### Syncytin-1 is overexpressed in HCC tissues

**Fig. 1:**
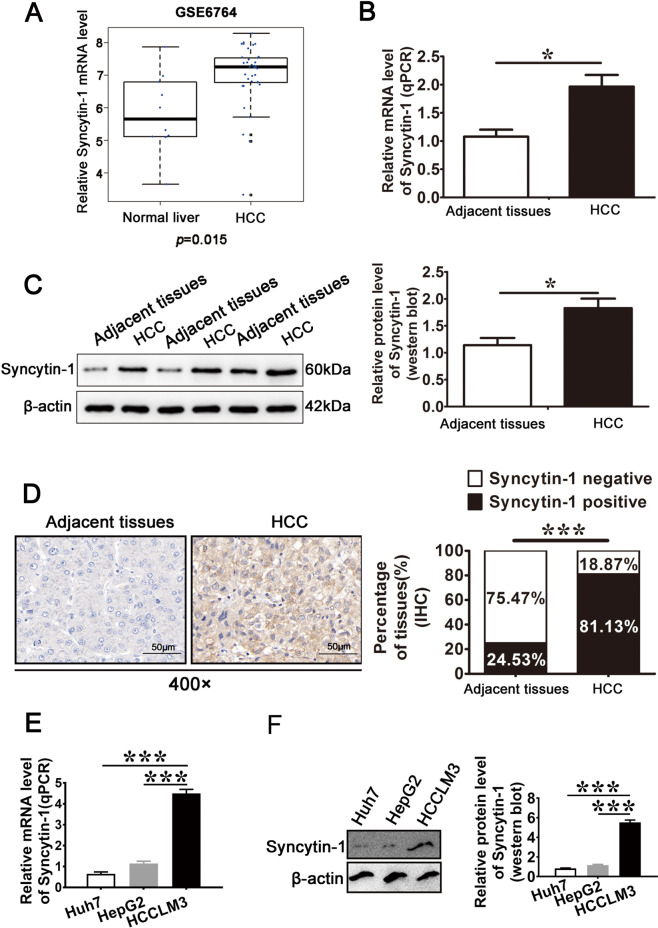
Syncytin-1 is highly expressed in HCC tissues and cell lines. **a** Data from GSE6764 was used to analyze the mRNA level of Syncytin-1 in HCC and normal liver. **b** The mRNA level of Syncytin-1 was determined by quantitative real-time PCR (qPCR) in HCC and adjacent tissues. **c** Syncitin-1 protein levels in 33 pairs of HCC and adjacent tissues were assessed using western blotting. **d** 53 paired of formalin-fixed HCC specimens and 50 formalin-fixed HCC without adjacent tissues (totally 103) were obtained to detect Syncytin-1 expression by IHC. Representative images are shown (magnification, ×400). **e** and **f** Syncytin-1 mRNA and protein levels in several HCC cell lines were investigated using qPCR (**e**) and western blotting (**f**), respectively. Each bar represents results from three independent experiments. *p < 0.05, ***p < 0.001.

To determine the expression level of Syncytin-1 in HCC, bioinformatics analysis using Gene Expression Omnibus (GEO) data (GSE6764) was performed [24]. The result indicated that the expression of Syncytin-1 was significantly higher in HCC tissues than that in normal liver tissues (Fig. 1a). We also collected and determined HCC samples and their corresponding NTs to confirm the bioinformatics results. Quantitative real-time PCR indicated that the mRNA level of Syncytin-1 was ~1.8-fold higher in HCC compared to adjacent tissues (*p* < 0.05, Fig. 1b). Increased mRNA level of Syncytin-1 was observed in 28 of 33 specimens (84.85%) and 7 out of 33 adjacent tissues (21.21%) (*p* < 0.001, Supplementary Table S1). The protein level of Syncytin-1 was about 1.4-fold higher in HCC compared to adjacent tissues (*p* < 0.05, Fig. 1c). Overexpression of Syncytin-1 was observed in 27 of 33 (81.82%) of HCC samples, but only in 8 of 33 (24.24%) of adjacent tissues by western blotting (*p* < 0.001, Supplementary Table S2). Data of immunohistochemistry (IHC) also showed that 84 out of 103 (81.55%) HCC specimens were positively stained, whereas only 13 out of 53 (24.53%) adjacent tissues were positive for Syncytin-1 (*p* < 0.001; Fig. 1d, Supplementary Table S3).

The expression level of Syncytin-1 was also determined in three HCC cell lines by quantitative real-time PCR and western blotting. As shown in Fig. 1e and f, Syncytin-1 was found to be expressed in all the HCC cell lines. Its expression level was higher in HCCLM3, which possessed a higher metastatic ability. Taken together, these data indicated that the expression of Syncytin-1 was increased in HCC.

### High expression of Syncytin-1 correlates with higher stages of HCC and predicts poor prognosis in HCC patients

**Fig. 2:**
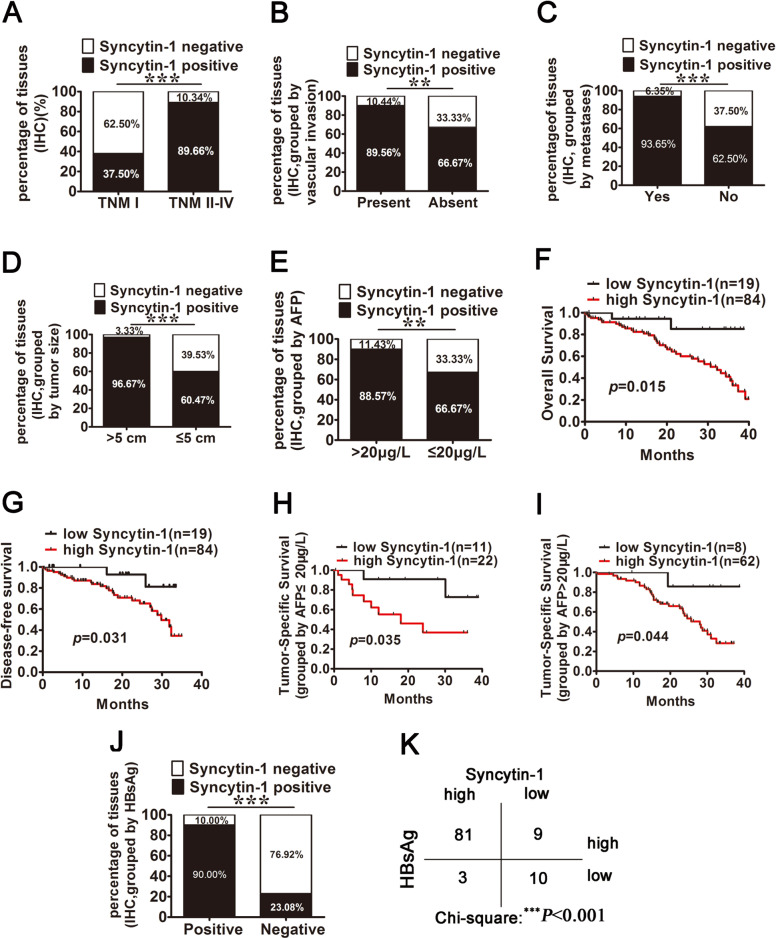
High expression of Syncytin-1 protein predicts advanced HCC and poor prognosis. The IHC data of Syncytin-1 expression were reanalyzed. **a** The expression of Syncytin-1 was analyzed in four stages of HCC tissues. **b**–**e** The positivity rate of Syncytin-1 expression in HCC with vascular invasion (**b**), metastases (**c**), different tumor sizes (**d**), and serum level of AFP (**e**) were analyzed. **f**–**i** Syncytin-1 overexpression was positively correlated with poor overall survival (**f**) and poor disease-free survival (**g**). HCC with high Syncytin-1 expression had a lower median overall survival than HCC without the Syncytin-1 overexpression, regardless of the absence (**h**) or presence (**i**) of high serum AFP. **j** and **k** The overexpression of Syncytin-1 was positive in correlation with serum HBsAg positivity. *p < 0.05, **p < 0.01, ***p < 0.001.

To further investigate the role of Syncytin-1 in the progression of HCC, the IHC data were reanalyzed in four stages of HCC tissues. Among the 87 HCC tissues at stages II–IV, 78 (89.66%) showed positive staining of Syncytin-1, whereas only 6 of 16 (37.50%) HCC tissues at stage I were Syncytin-1 positive (*p* < 0.001; Fig. 2a, Table 1). Therefore, the expression of Syncytin-1 was positively correlated with higher stages of HCC. Since the late-stage HCC presented greater invasive and metastatic potential [25], this result suggested that Syncytin-1 might promote the malignant progression of HCC.

**Table 1:** Univariate and multivariate analysis Syncytin-1 overexpression and various risk factors in 103 HCC patients.

Characteristic	Total	Univariate analysis				Multivariate analysis		
	*N* = 103	Syncytin-1 expression (−)	Syncytin-1 expression (+)	*χ*^2^	*p*	OR	95% CI	*p*
*Gender*				0.009	0.926			NA
Male	66	12(18.18)	54(81.82)					
Female	37	7(18.92)	30(81.08)					
*Age (years)*				5.213	0.022*	2.286	0.652–8.623	0.153
≤59	60	16(26.67)	44(73.33)					
>59	43	3(6.98)	40(93.02)					
*TNM*				24.794	<0.001***	10.357^a^	3.081–48.623	<0.001***
I	16	10(62.50)	6(37.50)					
II	33	4(12.12)	29(87.88)					
III	36	4(11.11)	32(88.89)					
IV	18	1(5.56)	17(94.44)					
*Metastases*				13.779	<0.001***	5.382	2.208–16.125	<0.001***
Yes	63	4(6.35)	59(93.65)					
No	40	15(37.50)	25(62.50)					
*Tumor size (cm)*				19.481	<0.001***	8.135	2.574–25.672	<0.001***
≤5	43	17(39.53)	26(60.47)					
>5	60	2(3.33)	58(96.67)					
*Microvascular invasion*				8.153	0.004**	4.017	1.264–10.683	0.005**
Present	67	7(11.67)	60(89.55)					
Absent	36	12(33.33)	24(66.67)					
*HBsAg*				29.515	<0.001***	12.065	3.818–82.067	<0.001***
Positive	90	9(10.00)	81(90.00)					
Negative	13	10(76.92)	3(23.08)					
*AFP*				7.153	0.007**	3.862	1.167–9.853	0.007**
>20 μg/L	70	8(11.43)	62(88.57)					
≤20 μg/L	33	11(33.33)	22(66.67)					

*NA* not adopted.

*P < 0.05, **p < 0.01, ***p < 0.001.

^a^The OR represents TNM stages II–IV compared to stage I.

We further determined the relationship between Syncytin-1 expression and the other clinicopathologic features using univariate and multivariable Cox regression analysis. Our results showed that the level of Syncytin-1 in HCC was positively correlated with vascular invasion (*p* = 0.004, Fig. 2b, Table 1) and metastasis (*p* < 0.001, Fig. 2c, Table 1). Since the risk of microvascular invasion and metastasis in HCC increases accompanying larger tumor size [26], we also analyzed the overexpression of Syncytin-1 in HCC with different tumor sizes. The overexpression of Syncytin-1 was significantly related to larger tumor size (>5 cm, *p* < 0.001, Fig. 2d, Table 1). Serum α-fetoprotein (AFP) levels are also a prognostic indicator [27, 28], which may affect prediction, diagnosis, and postoperative prognosis. Our results showed that overexpression of Syncytin-1 occurred more commonly in patients with higher serum AFP levels (>20 μg/L, *p* = 0.007) (Fig. 2e, Table 1). The Syncytin-1 expression did not correlate with gender and age using multivariable Cox regression analysis.

Survival analysis of our clinical samples showed that overexpression of Syncytin-1 was positively correlated with poor overall survival (OS, *p* = 0.015, Fig. 2f) and poor disease-free survival (DFS, *p* = 0.031, Fig. 2g). Further study revealed that HCC with high Syncytin-1 expression level had lower median overall survival than HCC without the Syncytin-1 overexpression, regardless of the absence or presence of high serum α-fetoprotein (AFP, Fig. 2h, i). Taken together, it suggested that HCC with a high expression level of Syncytin-1 was prone to have a poor clinical outcome.

### The level of Syncytin-1 is significantly associated with HBsAg-positive HCC patients

The molecular mechanisms underlying HBV-induced tumorigenesis remain debated. Our previous research manifested that HBx could induce overexpression of Syncytin-1 through NF-κB signal in HepG2 cells [14], suggesting Syncytin-1 may serve as a potential cofactor in HBV-related HCC. However, the relationship between Syncytin-1 expression and HBV-induced HCC still needs further investigation. As a result, we analyzed the ratio of Syncytin-1 overexpression in HCC patients with or without serum HBsAg positivity. Interestingly, 81 out of 90 (90.00%) HBsAg-positive HCC patients showed a high protein level of Syncytin-1, while Syncytin-1 overexpression was found only in 3 of 13 (23.08%) HBsAg-negative HCCs (Fig. 2j, Table 1). The ratio of Syncytin-1 overexpression in HCC patients with serum HBsAg positivity was ~3.90 times compared to that of HBsAg-negative HCCs.

In turn, we also evaluated the ratio of serum HBsAg positivity in HCC patients with or without Syncytin-1 overexpression. 81 out of 84 (96.43%) Syncytin-1-positive HCC patients displayed HBsAg positivity in serum, whereas serum HBsAg positivity was found only 9 of 19 (47.37%) in Syncytin-1-negative HCCs (Fig. 2k). Results from the study indicated that the incidence that HCC patients have Syncytin-1 and HBsAg simultaneously expression or not was up to ~88.35% (91/103) (Fig. 2k, Table 1), higher than co-expression incidence that Syncytin-1 with any other clinicopathological parameters. These results suggested a marked consistency between Syncytin-1 expression and HBsAg level.

The multivariate Cox regression test also manifested that HBsAg was positively correlated with Syncytin-1 expression (odds ratio = 12.065, *p* < 0.001, Table 1) in HCC. Spearman rank correlation analysis confirmed that there was a significant positive correlation between the expression of Syncytin-1 and serum HBsAg positivity (*r* = 0.573, *p* < 0.001, Table 2). From the above findings, the enhanced expression of Syncytin-1 might contribute to the progression of HBV-induced HCC.

**Table 2:** Spearman rank correlation analysis of the correlation between Syncytin-1 overexpression and various risk factors in 103 HCC patients.

Characteristic	Syncytin-1 positive	
	*r*	*p*
HBsAg positive	0.573	<0.001^***^
AFP > 20 μg/L	0.264	<0.01^**^
Tumor size>5 cm	0.460	<0.001^***^
TNM II–IV	0.487	<0.001^***^
Microvascular invasion	0.281	<0.01^**^
Metastases	0.391	<0.001^***^

^**^p < 0.01, ^***^p < 0.001.

### Syncytin-1 promotes HCC tumorigenicity

**Fig. 3:**
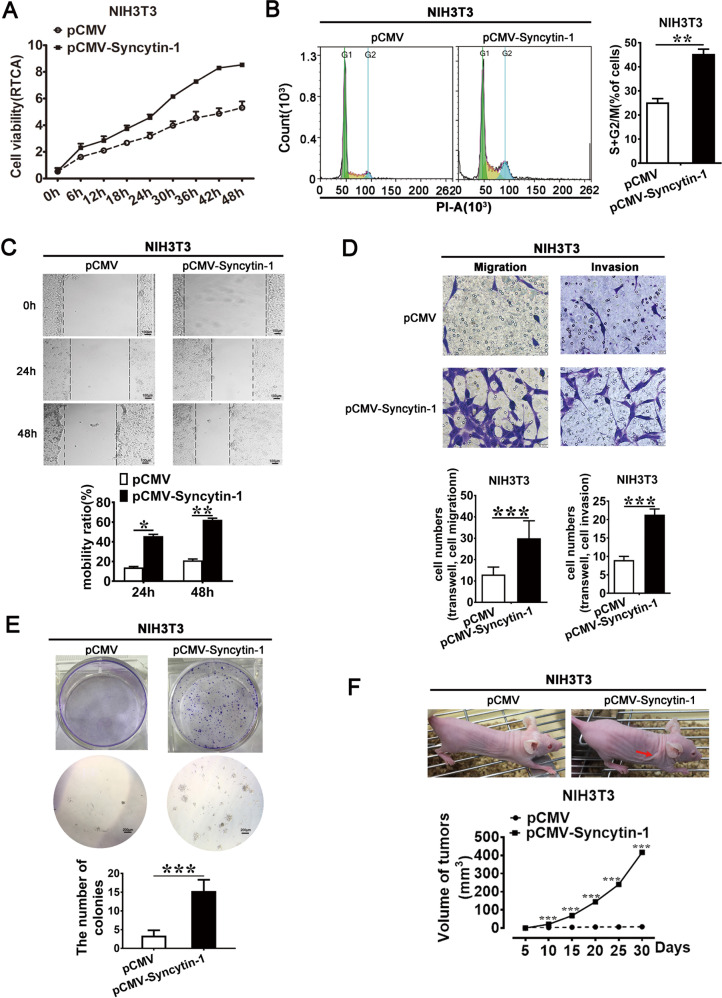
Syncytin-1 promotes cell marinization in NIH3T3 cells. **a** The effect of Syncytin-1 on cell proliferation was examined by RATC assay. **b** Variation of the cell cycles in Syncytin-1 overexpressed NIH3T3 cells was determined by flow cytometry. **c** The effect of Syncytin-1 on cell migration ability was assessed using the wound healing assay. **d** The effects of Syncytin-1 on cell migration and invasion were detected. **e** Foci formation assay was used to reveal the role of Syncytin-1 on cell transformation in NIH3T3. **f** Tumor xenograft assay. Arrows indicate the formation of xenograft tumors in nude mice. The graph shows the mean ± SEM of tumor volume induced by Syncytin-1-transfected NIH3T3 cells. All graphs represent at least three independent experiments. ***p < 0.001.

To determine the role of Syncytin-1 in HCC tumorigenicity, a series of experiments to detect cell proliferation, cell cycle progression, cell migration, and invasion, and cell malignant transformation were performed. HCC cell lines and NIH3T3, one of the most frequently used cell lines to explore the potential role of an oncogene on cell proliferation and migration in HCC [29, 30], were used. As shown in Fig. 3a, there was a time-dependent increase of cell viability in Syncytin-1-transfected NIH3T3 cells using a real-time cell analysis (RTCA) assay. Knockdown of Syncytin-1 expression in HCCLM3 cells by transfecting with pSilencer-shSyncytin-1 confirmed the above result (Supplementary Fig. S1a). Correspondingly, the results of cell cycle analysis also verified that Syncytin-1 increased the cell numbers of S and G2/M stages in both NIH3T3 and HCCLM3 cells (*p* < 0.01, Fig. 3b, Supplementary Fig. S1b). These results suggested that Syncytin-1 could promote proliferation and cell cycle progression in HCC cells.

The metastatic potential of Syncytin-1 was determined by wound healing assay and transwell. The results of the wound healing assay demonstrated that Syncytin-1 improved the mobility of NIH3T3 cells compared to control (*p* < 0.01, Fig. 3c). Conversely, knockdown of Syncytin-1 by pSilencer-shSyncytin-1 transfection inhibited the wound closure of HCCLM3 cells (*p* < 0.01, Supplementary Fig. S1c). The transwell migration assay and invasion assay with matrigel (*p* < 0.001, Fig. 3d, Supplementary Fig. S1d) confirmed the positive effect of Syncytin-1 on cell migration and invasion, respectively. The results above demonstrated that Syncytin-1 might play a vital role in tumor metastasis.

The function of Syncytin-1 on tumorigenic ability in HCC cells was also investigated. Foci formation assay revealed that NIH3T3-Syncytin-1 cells formed colonies ~5-fold higher than the control (*p* < 0.001, Fig. 3e). After knocking down Syncytin-1 in HCCLM3, the colony-forming ability was almost 75% decreased compared to the control (*p* < 0.001, Supplementary Fig. S1e). Furthermore, tumor xenograft assay showed that Syncytin-1 overexpression in NIH3T3 cells could induce tumor formation at the flank of nude mice, whereas down-regulation of Syncytin-1 expression in HCCLM3 cells effectively inhibited tumor formation (*p* < 0.001, Fig. 3f, Supplementary Fig. S1f, Supplementary Tables S4, 5). These results implicated that Syncytin-1 had oncogenic potential.

The above results indicated that Syncytin-1 could promote the development and progression of HCC.

### Syncytin-1 activates the MEK/ERK signal pathway in HCC

**Fig. 4:**
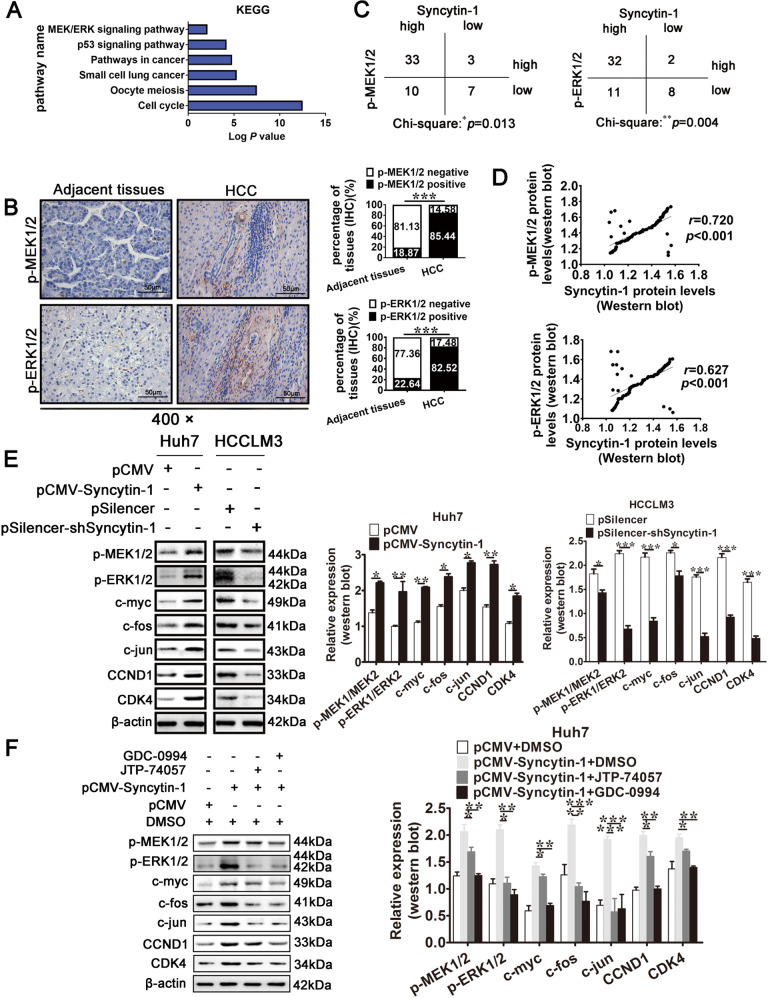
Syncytin-1 activates MEK/ERK pathway in HCC. **a** The upregulated genes in HCC were analyzed by KEGG analysis. **b** and **c** The expression of p-MEK1/2 and p-ERK1/2 in 53 pairs of formalin-fixed HCC and adjacent tissues were detected using IHC. Representative images are shown (magnification, ×400). **d** The expression of p-MEK1/2 and p-ERK1/2 were determined using western blotting. The correlation between the expression of Syncytin-1 and p-MEK1/2 or p-ERK1/2 level was analyzed using Pearson’s correlation test. **e** The levels of p-MEK1/2, p-ERK1/2, and their downstream proteins (c-myc, c-fos, c-jun, CCND1, and CDK4) were examined in HCC cell lines transfected with Syncytin-1 or pSilencer-shSyncytin-1 by western blotting. **f** The levels of p-MEK1/2, p-ERK1/2, and their downstream proteins were detected after using MEK/ERK-specific inhibitors (JTP-74057, or GDC-0994) in Huh7 transfected with Syncytin-1. The bars represent results from at least three independent experiments. *p < 0.05, **p < 0.01, ***p < 0.001.

Recent studies revealed that the inflammation-activated MEK/ERK pathway was involved in the development of several types of cancers [22, 31, 32]. Bioinformatics analyses using GSE41804 [33] suggested that the genes correlated with HCC were involved in the MEK/ERK pathway, cell cycle, and other pathways in cancer (Fig. 4a, Supplementary Fig. S2a, b, and Supplementary Tables S6, 7). An in-depth analysis showed that the patients with high expression levels of MEK1, ERK2, and its downstream gene cyclin D1 (CCND1) were more likely to be associated with the poor OS (Supplementary Fig. S2c–e). Overexpression of ERK1 and the downstream protein cyclin-dependent kinase4 (CDK4) correlated with vascular invasion (Supplementary Fig. S2, f, g). In silico results indicated that MEK/ERK pathway was implicated in the poor progression of HCC.

To further determine whether MEK/ERK pathway was activated in HCC, we detected the phosphorylation level of MEK1/2 (p-MEK1/2) and ERK1/2 (p-ERK1/2) in paired HCC tissues using IHC. As displayed in Fig. 4b, significantly increased p-MEK1/2 (*p* < 0.001) and p-ERK1/2 (*p* < 0.001) levels were found in HCC specimens. These results suggested that MEK/ERK pathway might be activated in HCC tissues. Since accumulation evidence showed that Syncytin-1 could regulate inflammatory abnormalities [5, 34], it is speculated that Syncytin-1 might aberrantly regulate the inflammation-activated pathway in HCC. Interestingly, our clinical analysis indicated that the positive rate of p-MEK1/2 and p-ERK1/2 were significantly higher in Syncytin-1-positive patients than those in Syncytin-1-negative patients (Fig. 4c). To examine the potential relationship between Syncytin-1 and p-MEK/p-ERK, we analyzed the relationship between the expression levels of Syncytin-1 and p-MEK/p-ERK by Pearson correlation analysis. The results displayed a linear regression between Syncytin-1 and p-MEK1/2 (*r* = 0.720, *p* < 0.001) or p-ERK1/2 in HCC patients (*r* = 0.627, *p* < 0.001, Fig. 4d), suggesting that the expression of Syncytin-1 was positively related to the level of p-MEK/p-ERK.

Studies were then performed to assess the effect of Syncytin-1 on the MEK/ERK signal pathway in HCC cell lines. As shown in Supplementary Fig. S3a, western blot analysis revealed the overexpression of Syncytin-1 after transfected with pCMV-Syncytin-1 plasmids in Huh7 cells. The knockdown of Syncytin-1 in HCCLM3 cells was indicated in Supplementary Fig. S3b. It was shown that Syncytin-1 could increase the expression of p-MEK1/2, p-ERK1/2, and its downstream genes, including CCND1, CDK4, c-myc, c-fos, and c-jun in Huh7 cells, while knockdown of Syncytin-1 resulted in a decrease of the MEK/ERK pathway proteins in the HCCLM3 cell line (Fig. 4e). However, Syncytin-1 did not affect the expression of total MEK1/2 and ERK1/2 (Supplementary Fig. S3a, b). To further confirm whether Syncytin-1 upregulated these downstream proteins through activating the MEK/ERK pathway in HCC cells, JTP-74057, an inhibitor of MEK1/2 activity [35], and GDC-0994, a highly selective ERK1/2 inhibitor [36], were used. After transfection of Syncytin-1 in Huh7 cells, the level of Syncytin-1 was significantly increased (Supplementary Fig. S3c). The results showed that JTP-74057 or GDC-0994 treatment effectively reversed the expression of p-MEK1/2, p-ERK1/2, and their downstream proteins (Fig. 4f), without changing of total MEK1/2 and ERK1/2 expression, in Syncytin-1-transfected Huh7 cells (Supplementary Fig. S3c). In conclusion, our results indicated that Syncytin-1 activated MEK/ERK signal pathway in HCC.

### Syncytin-1 enhances HCC tumorigenicity through MEK/ERK pathway

**Fig. 5:**
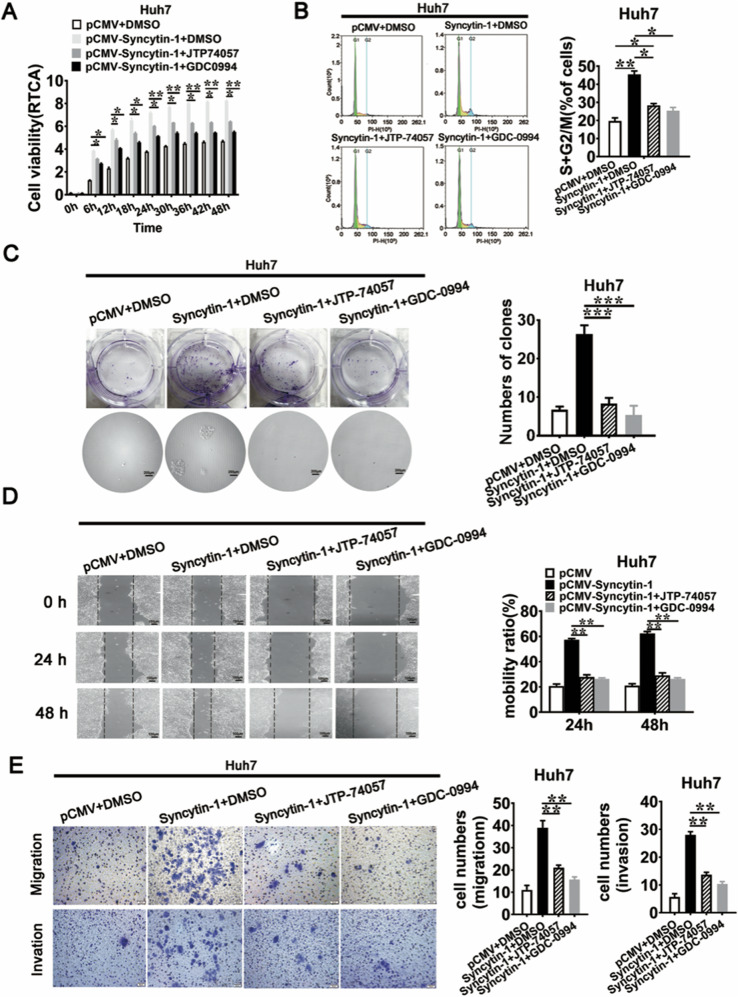
MEK/ERK signal pathway is necessary for Syncytin-1 induced malignant properties in HCC cells. Huh7 cells transfected with Syncytin-1 were treated with JTP-74057 or GDC-0994, which were specific for MEK/ERK pathway inhibitors. **a** RTCA assay was used to detect the role of Syncytin-1 on cell proliferation when blocking MEK/ERK pathway. **b** Flow cytometry. MEK/ERK signal pathway regulation of Syncytin-1-induced cell cycle progression was demonstrated. **c** The effect of the MEK/ERK pathway on Syncytin-1-induced cell transformation was determined. **d** and **e** The role of the MEK/ERK pathway on Syncytin-1-regulated cell migration or invasion was investigated using wound healing assay (**d**), transwell migration assay, and transwell invasion assay (**e**). All graphs show at least three independent experiments. *p < 0.05, **p < 0.01, ***p < 0.001.

The above results showed that Syncytin-1 could induce malignant properties in HCC, as well as activate the MEK/ERK signal pathway. Combined with the MEK/ERK pathway was positively correlated with HCC progression, we speculated that the MEK/ERK signal might play a critical role in Syncytin-1-induced HCC carcinogenesis and metastasis. To prove this hypothesis, specific MEK inhibitor JTP-74057 and ERK inhibitor GDC-0994 were used. RTCA showed that both inhibitors could induce a time-dependent decrease of cell viability in Huh7-Syncytin-1 cells (Fig. 5a). The proportion of Huh7-Syncytin-1 cells in S and G2/M phases was decreased after treatment with both inhibitors (Fig. 5b). These results indicated that Syncytin-1 promoted HCC cell proliferation through MEK/ERK pathway.

To investigate whether Syncytin-1 induced cell malignancy tumorigenesis in HCC via MEK/ERK pathway, foci formation assay was used. As displayed in Fig. 5c, Syncytin-1 induced colony formation was reversible under the treatment of JTP-74057 or GDC-0994 in Huh7 (*p* < 0.001).

Our results also showed that MEK/ERK inhibitors effectively inhibited Syncytin-1-mediated cell migration by wound healing assay (*p* < 0.01, Fig. 5d) and transwell migration assay (*p* < 0.01, Fig. 5e). Transwell invasion assay revealed that both MEK/ERK inhibitors effectively suppressed cell invasion in Huh7–pCMV–Syncytin cells (*p* < 0.01, Fig. 5e). In conclusion, we demonstrated that Syncytin-1 could enhance tumor malignant transformation and metastasis via the MEK/ERK pathway in HCC.

### Syncytin-1 prevents doxorubicin-induced apoptosis via MEK/ERK cascade

**Fig. 6:**
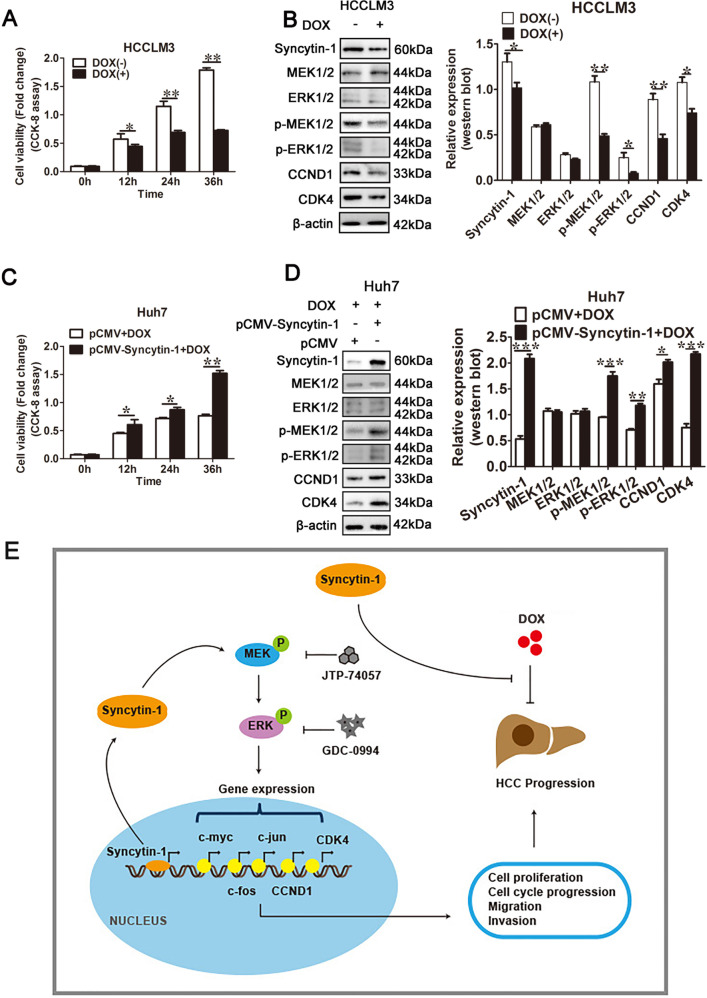
Syncytin-1 promotes doxorubicin resistance via MEK/ERK pathway in HCC. **a** CCK-8 assay. The effect of doxorubicin on cell viability was determined in HCCLM3 cells. **b** The regulation of doxorubicin on the expression of MEK/ERK pathway protein (MEK1/2, ERK1/2, p-MEK1/2, p-ERK1/2, CCND1, and CDK4) were detected by western blotting. **c** CCK-8 assay was used to investigate the role of Syncytin-1 on doxorubicin-induced cell apoptosis in Huh7 cells. **d** The function of Syncytin-1 overexpression on doxorubicin suppressed MEK/ERK pathway activation was assessed using western blotting. **e** Schema illustrates the mechanism by which Syncytin-1 promotes the development of HCC and doxorubicin resistance through activation of the MEK/ERK pathway. Syncytin-1 induced the activation of the MEK/ERK pathway and upregulation of its downstream proteins (such as c-myc, c-fos, c-jun, CCND1, and CDK4) levels in HCC. These genes participated in the malignant progression of HCC by promoting cell proliferation, migration, and invasion. Syncytin-1 also prevented HCC cells from doxorubicin-induced cell apoptosis. DOX, doxorubicin. *p < 0.05, **p < 0.01, ***p < 0.001.

Doxorubicin is a chemotherapy medication widely used to treat human cancers and mediate the cellular apoptotic effect [37]. Doxorubicin treatment significantly decreased the cell viability of HCCLM3 by cell counting kit-8 (CCK-8) assay (*p* < 0.01, Fig. 6a). Of interest, the protein level of Syncytin-1 was down-regulated with doxorubicin treatment (*p* < 0.05, Fig. 6b). What’s more, the levels of p-MEK, p-ERK, CCND1, and CDK4 were also markedly inhibited by doxorubicin treatment (Fig. 6b), suggesting that doxorubicin treatment could suppress the expression of Syncytin-1 and the activation of the MEK/ERK pathway.

To determine the role of Syncytin-1 on doxorubicin-mediated HCC cell apoptosis, Huh7 cells, which with the lower expression level of Syncytin-1, were transfected with pCMV-Syncytin-1 plasmids followed by treatment with doxorubicin. The CCK-8 assay showed that Syncytin-1 induced a significant time-dependent increase of cell viability compared to control in doxorubicin-treated Huh7 cells (*p* < 0.01, Fig. 6c). Additionally, Syncytin-1 increased the levels of p-MEK (*p* < 0.001), p-ERK (*p* < 0.01), CCND1 (*p* < 0.05), and CDK4 (*p* < 0.001), without changing of total MEK1/2 and ERK1/2 expression, in doxorubicin-treated Huh7 cells (Fig. 6d). These results suggested that Syncytin-1 might inhibit doxorubicin-induced apoptosis via MEK/ERK cascade.

## Discussion

HCC is one of the most common malignancies worldwide. Despite therapeutic advances, patients with HCC still have a poor survival rate [38]. The presence of microvascular invasion and extrahepatic metastasis leading to progressive development is one of the major causes for its dismal clinical outcome [39, 40]. To improve the survival rate in HCC patients, it is urgent to investigate the biomarkers for the prognosis of HCC with metastasis. In this study, we found that Syncytin-1 might be an independent biomarker of metastatic invasiveness in HCC.

Syncytin-1 was overexpressed in HCC compared to adjacent tissues (Fig. 1), indicating a strong correlation between HCC and the expression level of Syncytin-1. Tumor-node-metastasis (TNM) stage, tumor size, microvascular invasion, and metastasis are prognostic factors in HCC [41]. Our further bioinformatics and clinical data analysis showed that Syncytin-1 overexpression was related to the TNM stage, vascular invasion, metastasis, tumor size (Fig. 2a–e). Additionally, HCC patients with a higher level of Syncytin-1 were more likely associated with worse clinical outcomes (Fig. 2f, g). Taken together, it suggested that Syncytin-1 might contribute to HCC progression. Serum AFP level is usually used to help diagnose patients with HCC [42, 43]. It was interesting that the overexpression of Syncytin-1 was highly related to poor OS, regardless of with or without high serum AFP levels in HCC (Fig. 2h, i), suggesting that Syncytin-1 might be a novel potential diagnostic biomarker for HCC.

Chronic HBV infection is the leading cause of HCC. However, only a minority of HBV carriers eventually develop HCC, suggesting the presence of important cofactors in HBV-related HCC [44]. Our previous study has indicated that HBx can act as an oncogene and induce overexpression of Syncytin-1 through NF-κB in HCC cell lines [21], implying Syncytin-1 might involve in HBV-related HCC. In this study, we showed that Syncytin-1 was significantly correlated with serum HBsAg positivity (Fig. 2j, k). These results strongly indicated that Syncytin-1 might be a cofactor of HBV in the development of HCC.

Studies have reported that Syncytin-1 promoted proliferation and tumorigenesis in several types of cancers [13–16]. To investigate the effect of Syncytin-1 in HCC, several malignant properties of NIH3T3 and HCC cell lines were explored. Our data demonstrated that Syncytin-1 could induce cell proliferation and promote cell cycle progression at the G2/M phase. The wound-healing assay and transwell assay revealed that Syncytin-1 could enhance migration and invasion. Furthermore, foci formation assay and tumor xenograft assay suggested that Syncytin-1 enhanced the tumorigenesis in cell lines (Fig. 3, Supplementary Fig. S1). All these results implicated Syncytin-1 might promote the oncogenic potential of HCC.

Syncytin-1, which served as an immunotoxin, modulates inflammatory cascades [5, 34, 45–47]. Combined with the results that Syncytin-1 had oncogenic potential in HCC (Figs. 1–3), it was highly suggested that Syncytin-1 might promote HCC development through an aberrantly regulated inflammation-activated signal pathway. Bioinformatics prediction and clinical analysis revealed that the MEK/ERK pathway, involved in cancer progression, was activated in HCC (Fig. 4a-b). Further studies indicated that the expression of Syncytin-1 positively correlated with pMEK/pERK level (Fig. 4c, d). We further determined the role of Syncytin-1 on the expression of MEK/ERK pathway proteins. The western blotting analysis showed that the phosphorylation level of MEK1/2, ERK1/2, and the protein level of a series MEK/ERK pathway proteins, including c-myc, c-fos, c-jun, CDK4, and CCND1, were upregulated (Fig. 4e), implying that Syncytin-1 upregulated the expression of MEK/ERK pathway proteins in HCC. Since the downstream proteins had cross-talk with other signal pathways, we further determined whether Syncytin-1 regulated these downstream proteins through MEK/ERK pathway. Two small molecule inhibitors specific to MEK/ERK, JTP-74057, and GDC-0994, were used to block MEK/ERK signal pathway. Our results showed that Syncytin-1-induced upregulation of MEK/ERK and its downstream proteins was suppressed by JTP-74057 or GDC-0994 (Fig. 4f), suggesting that Syncytin-1 regulated the expression of CDK4, CCND1, c-myc, c-fos, and c-jun via the MEK/ERK.

CDK4 and CCND1 are key cell cycle-related genes in controlling cell proliferation [48]. C-myc, c-fos, and c-jun are all proto-oncogenes, involved in the initiation and regulation of oncogenic progression [49–51]. The upregulation of these proteins by the MEK/ERK pathway might contribute to Syncytin-1-induced HCC. Our results have shown that Syncytin-1-induced proliferation and cell cycle progression were reversed after treatment with MEK/ERK pathway inhibitors (Fig. 5a, b). Syncytin-1 also failed to promote migration, invasion, and tumorigenesis in HCC cells when blocking the MEK/ERK pathway (Fig. 5c–e). In summary, this study indicated that Syncytin-1 might enhance carcinogenesis and tumor metastasis in HCC through the MEK/ERK pathway. MEK/ERK pathway can mediate epithelial-to-mesenchymal transition in cancer [52], which is also the potential mechanism that leads to HCC progression.

Doxorubicin is a chemotherapy medication to treat cancer by inducing DNA damage and cell apoptosis [37]. After the treatment of doxorubicin, the viability of HCC cells decreased dramatically, accompanied by significantly down-regulation of Syncytin-1 expression level and the activity of the MEK/ERK pathway (Fig. 6a, b). However, overexpression of Syncytin-1 increased cell viability and reverted the inhibition of MEK/ERK cascade mediated by doxorubicin compared to control (Fig. 6c, d). These data indicated that Syncytin-1 might induce doxorubicin resistance in HCC cells through the MEK/ERK signal pathway.

In conclusion, our results showed that Syncytin-1 was highly expressed in HCC tissues, and related to advanced HCC. Syncytin-1 was a risk factor, which independent of serum AFP levels, to predict vascular invasion and poor prognosis in HCC patients. Syncytin-1 was also a potential cofactor in HBV-induced HCC. Syncytin-1 might trigger hepatocarcinogenesis and doxorubicin-resistance via the inflammation-activated MEK/ERK pathway (Fig. 6e). These results might provide novel insights into the mechanism underlying the development of HCC, as well as put forward potential therapeutic strategies of HCC.

## Materials and methods

### Bioinformatics analysis

The Oncomine database (http://www.oncomine.org/resource/login.html) was used to predict the expression of Syncytin-1 in HCC. The differentially expressed genes (DEGs) between the HCC and NT were identified using the R programming language’s limma package. Studies were performed using the GEO (http://www.ncbi.nlm.nih.gov/geo) human HCC microarray dataset with accession numbers GSE6764 [24] and GSE41804 [33] in the GPL570 platform (Affymetrix Human Genome U133 Plus 2.0 Array). With the standard of |logFC| ≥ 1 and *p*-value < 0.05, all statistical analyses were performed with R software. The R software was also used to analyze differential gene expression patterns between tumor and normal samples. Gene Ontology (GO) analysis was conducted to demonstrate the functions of the target genes in the biological process, cellular component, and molecular function. Kyoto Encyclopedia of Genes and Genomes (KEGG) pathway analysis was used to excavate remarkable pathways associated with target genes.

### Clinical samples

Human HCC tissues and the adjacent tissues (*n* = 33) were collected for quantitative real-time RT-PCR and western blotting analysis. Another 53 paired of formalin-fixed HCC specimens and 50 formalin-fixed HCC without adjacent tissues were obtained for IHC staining. All samples were collected from the patients who underwent surgical resection in Renmin Hospital, Wuhan University, and signed informed consent before their operations. The clinicopathological data of patients were obtained. The 8th edition of the TNM staging system, jointly developed by the American Joint Commission on Cancer (AJCC) and the Union for International Cancer Control (UICC), was used to classify the samples. Sample collections were approved by the Ethics committee of Wuhan University, School of Medicine (Wuhan, China). The study was conducted following the International Ethical Guidelines for Biomedical Research Involving Human Subjects (CIOMS).

### Plasmid construction and transfection

Plasmid pCMV-Syncytin-1 was established in our laboratory as described previously [53]. One short hairpin RNA targeting Syncytin-1 (sequence: GGTAACTCCTCCCACACAA) and (control) was cloned into the pSilencer 2.1-U6 neo Vector.

Cells were transiently transfected with indicated plasmid DNA for 48 h using TurboFect Transfection Reagent (Thermo Fisher Scientific, Cleveland, USA) according to the manufacturer’s instructions. The corresponding empty vector (pCMV or pSilencer 2.1-U6 neo) was used as a negative control.

### Cell culture

HepG2 and NIH3T3 cell lines were purchased from the American type culture collection (ATCC, Manassas, USA). Huh7 cell line was obtained from the Japan Health Science Research Resources Bank (Tokyo, Japan). The HCCLM3 cell line was from the Cell Bank of the China Center for Type Culture Collection (Wuhan, China). The cell lines were cultured at 37 °C with 5% CO_2_ in Dulbecco’s Modified Eagle Medium (GIBCO, NY, USA) supplemented with 10% fetal bovine serum (GIBCO), 100 units/mL penicillin, and 0.1 mg/mL streptomycin. All experiments were performed with mycoplasma-free cells. The cell lines used in this study have been authenticated by STR profiling.

### Pharmacologic treatment

JTP-74057, a highly specific and potent MEK1/2 inhibitor [35], and GDC-0994, a highly selective ERK1/2 inhibitor [36], were used to prevent the activation of the MEK/ERK pathway. Cells were treated with 1 nmol/L JTP-74057 or GDC-0994 in a humidified incubator for 24 h at 37 °C with 5% CO_2_.

Doxorubicin is a chemotherapy medication used to treat cancer. Cells were treated with 5 μmol/L doxorubicin for 24 h, followed by the cell counting kit-8 (CCK-8) assay and western blotting.

### Quantitative real-time PCR

Total RNA was isolated using Trizol^®^ reagents (Invitrogen, Carlsbad, USA). After treating with RNase-free DNase (Promega, WI, USA) to degrade potential DNA contamination, 1 μg RNA was converted into first-stranded DNA by the Reverse Transcriptase ReverTra Ace ^TM^ (TOYOBO, Osaka, Japan). Quantitative real-time PCR was performed in the iCycler System (Bio-Rad, CA, USA) using the SYBR Green PCR master mix (Roch Diagnostics, Mannheim, Germany). The primers used in this study were as follows: Syncytin-1 (Gene ID: 30816) forward: 5′-CCAATGCATCAGGTGGGTAAC-3′, Syncytin-1 reverse: 5′-GAGGTACCACAGACAAAAAATATTCCT-3′. β-actin was used as an internal control for quantification. The results were given as 2^−ΔΔCt^ values.

### Western blotting

HCC tissues and cell lines were lysed using M-PER mammalian protein extraction reagent (Pierce, Thermo Fisher Scientific, Inc.) containing the protease inhibitors following the standard procedures. Protein samples (40 μg) were separated in 4–12% SDS–PAGE gel and transferred onto a nitrocellulose membrane (Amersham Pharmacia Biotech, San Francisco, CA). Western blotting was performed according to the manufacturer’s protocols. The following antibodies purchased from Abcam were used in this study: Syncytin-1 (1:2000, ab179693), MEK1/2 (1:5000, ab178876), p-MEK1/2 (S218 + S222) (1:1000, ab194754), c-fos (1:2000, ab208942), c-myc (1:1000, ab32072), c-jun (1:1000, ab31419), CCND1 (1:2000, ab40754), CDK4 (1:1000, ab108357), rabbit IgG (1:2000, ab6721), and β-actin (1:5000, ab227387). ERK1/2 (1:1000, A10613), and p-ERK1 (T202/Y204)/ERK2 (T185/Y187) (1:1000, AP0472) were from ABclonal Technology. β-actin was used as an internal control. The immunoreactive bands were visualized using an ECL reagent (Pierce) according to the manufacturer’s recommendation. The bands were quantified by densitometry with ImageJ software (U. S. National Institutes of Health, Bethesda, MD, USA).

### IHC

IHC staining was performed according to the standard procedures using the following antibodies: Syncytin-1 (1:200, Abcam, ab179693), p-MEK1/2 (S218 + S222) (1:200, Abcam, ab194754), and p-ERK1 (T202)/ERK2 (T185) (1:200, Abcam, ab201015) were used as primary antibodies. The secondary antibody was Biotinylated goat anti-rabbit IgG (1:1000, CWBIO, cw0156s). Staining intensity was graded as 0 (negative), 1 (weak), 2 (moderate), 3 (strong), and 4 (very strong). Positive samples were scored as 2+, 3+, or 4+. Scores of 0 and 1+ were considered negative.

### RTCA

The Real-Time Cell Analyzer (RTCA S16, xCELLigence, ACEA Biosciences, San Diego, CA, USA) was used as described previously to detect the real-time effects of Syncytin-1 on cell proliferation [54]. 5 × 10^3^ cells were seeded in each well plate with electrodes for 18 h, then treated with different conditions. The sensor analyzer automatically monitored continuously for up to 48 h and expressed as cell index, which represents the cell viability. Cell index values were normalized to the value at the beginning of the treatment.

### Flow cytometry

Approximately 1 × 10^6^ cells were harvested and fixed with ice-cold 70% ethanol overnight. The single-cell suspension was prepared and incubated with the staining solution containing 40 μg/mL propidium iodide and 100 μg/mL RNase A for 15 min at room temperature. Then the cell cycle was analyzed by the flow cytometer (FACSAria III, B.D. Bioscience, Breda, The Netherlands) in the Research Center for Medicine and Structural Biology, Wuhan University.

### Wound healing assay

The cells in 12-well plates were scratched using a pipette tip. Cell migration into the wound area was monitored and photographed at 0, 24, and 48 h using an inverted phase-contrast microscope (Olympus CH-40, Olympus, Tokyo, Japan). The migration rate was calculated as the proportion of the mean distance between borderlines caused by scratching to the distance, which remained cell-free after migration. Three representative images from each coverslip of the scratched areas under each condition were recorded and averaged.

### Transwell assay

For cellular migration assays, cells in serum-free media were seeded into 24-well Transwell^TM^ chambers (Costar, Cambridge, MA, USA) at 1 × 10^5^ cells/mL. Medium containing 10% FBS was added (500 µL/well) to the lower chambers to serve as the chemoattractant. Incubated at 37 °C for 48 h, the cells that had migrated through the membrane were fixed, stained with crystal violet, and then counted under a microscope (Olympus CH-40).

For cellular invasion assay, transwell with Matrigel (200 μg/mL, B.D. Biosciences, San Jose, CA, USA) was used. The other procedures were the same as above.

### Foci formation assay

The cells transfected with indicated plasmids were seeded in six-well plates with 1000 cells per well and incubated at 37 °C. After 2 weeks, the colonies were stained with crystal violet and examined under an Olympus CH-40 microscope (Olympus, Tokyo, Japan). The colony consisted of more than 50 cells were counted.

### Tumor xenograft assay

Xenograft tumor assay was used to determine the tumorigenic activity of Syncytin-1 in vivo. Cells were subcutaneously injected into the dorsal flank of BALB/C-nu mice (4–5 weeks old, 15–20 g weight, randomly divided into different groups, with 5 mice per control group and 8 mice per experimental group). Tumor volumes (mm^3^) were measured every 5 days after injection up to 30 days and calculated using the ellipsoid formula: 4/3 × *π*×(*L*/2 × *W*/2 × *H*/2). All animal care and handling procedures were performed according to the National Institutes of Health’s Guide for the Care and Use of Laboratory Animals. Animal experiments were approved by the Animal Ethics Committee of Wuhan University, Wuhan University Center for Animal Experiment/A3 Laboratory.

### CCK-8 assay

Approximately 5 × 10^3^ cells/well were seeded in 96-well plates with 100 μL medium each well. After indicated treatment and incubation, a 10 μL CCK-8 solution was added to each well. The plate was incubated for an additional 2 h before measuring the absorbance at 450 nm wavelength using a microplate reader.

### Statistical analysis

The data were from at least three independent randomized trials. All testing was done blind, in duplicate by two technicians. Statistical analyses were conducted using the R-Studio statistical software. Student’s *t*-test and one-way ANOVA were used to compare quantitative variables. The Chi-square test was used to analyze qualitative variables. Pearson’s correlation test was used to assess the correlation between variables with a normal distribution. Spearman’s rank correlation test was used to evaluate the correlation between rank variables. Kaplan–Meier analysis was used for survival analysis. Multivariate analyses were performed using the Cox proportional hazards model. Results were presented as mean ± standard error of the mean (SEM), or mean ± standard deviation (SD). *p* < 0.05 was considered to be significant.

## Supplementary information

Supplementary materials

Supplementary Fig. S1 Syncytin-1 promotes cell malignant properties in HCCLM3.

Supplementary Fig. S2 Inflammation-activated MEK/ERK pathway displays a robust positive correlation with HCC progression.

Supplementary Fig. S3 Syncytin-1 has no effect on total MEK1/2 and ERK1/2 protein.

## Data Availability

The datasets used and analyzed in this study are available from the corresponding author on reasonable request.
