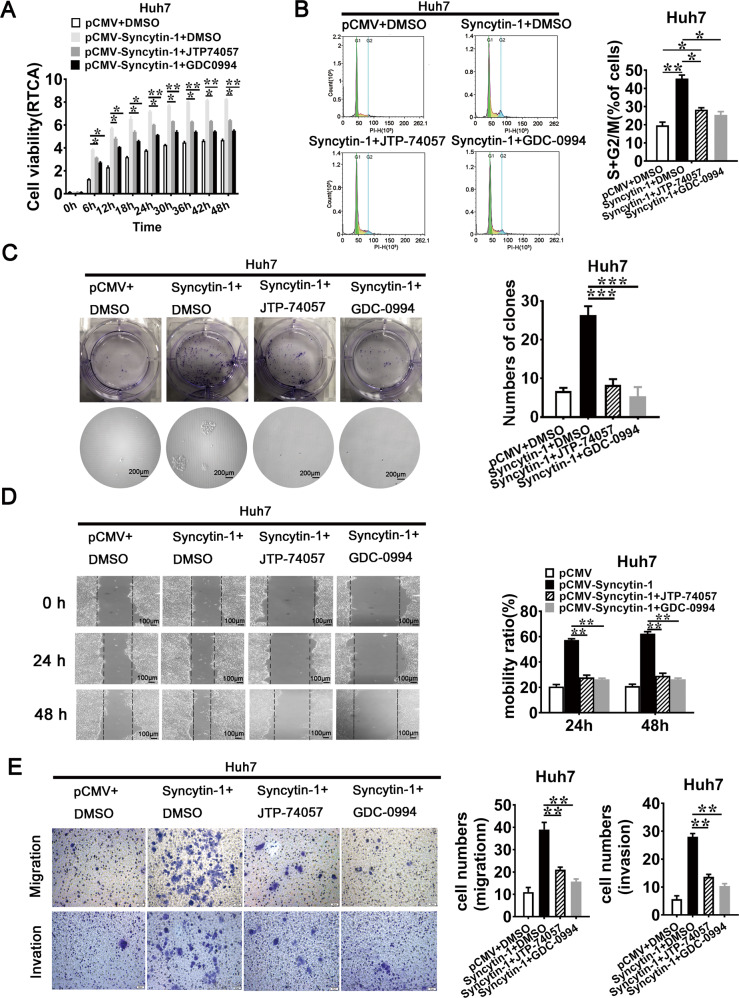# Correction to: Implication of human endogenous retrovirus W family envelope in hepatocellular carcinoma promotes MEK/ERK-mediated metastatic invasiveness and doxorubicin resistance

**DOI:** 10.1038/s41420-022-00952-3

**Published:** 2022-04-01

**Authors:** Yan Zhou, Lijuan Liu, Youyi Liu, Ping Zhou, Qiujin Yan, Honglian Yu, Xiaobei Chen, Fan Zhu

**Affiliations:** 1 State Key Laboratory of Virology and Hubei Province Key Laboratory of Allergy & Immunology, Department of Medical Microbiology, School of Medicine, Wuhan University, 430071 Wuhan, P. R. China; 2 Wuxi School of Medicine, Jiangnan University, 214000 Wuxi, P. R. China; 3 Department of Biochemistry and Collaborative Innovation Center, Jining Medical University, 272067 Jining, P. R. China; 4 Department of Infectious Diseases, Renmin Hospital, Wuhan University, 430071 Wuhan, P. R. China

**Keywords:** Prognostic markers, Prognostic markers

Correction to: *Cell Death Discovery* 10.1038/s41420-021-00562-5, published online 08 July 2021

The original version of this article unfortunately contained a mistake. After publication of this article, the authors noticed an accidental duplication of Fig. 5E: the microscopy imagines of transwell for migration in the Syncytin-1+JTP-74057 and Syncytin-1+GDC-0994 groups. All text referring to the figure, including the legend, are correct and this does not impact the findings of the study. The corrected figure is supplied below. The authors apologize for the mistake. The original article has been corrected.