# Supplementary material for: Implication of human endogenous retrovirus W family envelope in hepatocellular carcinoma promotes MEK/ERK-mediated metastatic invasiveness and doxorubicin resistance
Source: Cell Death Discov. 2021 Jul 8;7:177. doi: 10.1038/s41420-021-00562-5 (PMC8266889; doi:10.1038/s41420-021-00562-5)
Supplement: Supplementary file 1 — Supplementary materials [file 41420_2021_562_MOESM1_ESM.docx]

**Supplementary Fig. S1 Syncytin-1 promotes cell malignant properties in HCCLM3. a** The effect of Syncytin-1 on cell proliferation in HCCLM3 was examined by RATC assay. **b** Variation of the cell cycles in Syncytin-1 downregulated HCCLM3 cells were determined by flow cytometry. **c** The effect of Syncytin-1 on cell migration ability was assessed using the wound healing assay. **d** The effects of Syncytin-1 on cell migration and invasion were detected. **e** Foci formation assay was used to reveal the role of Syncytin-1 on HCCLM3 cell transformation. **f** Tumor xenograft assay. Arrows indicate the formation of xenograft tumors in nude mice. The graph shows the mean±SEM of tumor volume induced by shSyncytin-1 transfected HCCLM3 cells. The experiments were repeated at least three times. **p*<0.05, ***p*<0.01, ****p*<0.001

**Supplementary Fig. S2 Inflammation-activated MEK/ERK pathway displays a robust positive correlation with HCC progression.** Bioinformatics analyses were used to show the role of the MEK/ERK pathway in HCC. **a-b** 1047 differentially expressed genes, including 511 up-regulated genes and 536 down-regulated genes, were identified in GSE41804 by using R software. **c** The relationship between MEK1 copy number and recurrence at 5 years of HCC in Guichard liver database. **d-e** Correlation analysis of ERK2 **(d)** or CCND1 **(e)** expression and overall survival of HCC in TCGA or Guichard liver cohort. **f-g** Role of ERK1 **(f)** and CDK4 **(g)** in the vascular invasion of HCC in Wurmbach liver database.

**Supplementary Fig. S3 Syncytin-1 has no effect on total MEK1/2 and ERK1/2 protein.** **a-b** The expression of Syncytin-1, MEK1/2, and ERK1/2 were determined using western blotting in pCMV-Syncytin-1 transfected Huh7 cells **(a)** or pSilencer-shSyncytin-1 transfected HCCLM3 cells **(b)**. **c** The levels of Syncytin-1, MEK1/2, and ERK1/2 were detected after using ERK/MEK specific inhibitors (JTP-74057, or GDC-0994) in Huh7 transfected with Syncytin-1. The bars represent results from at least three independent experiments. **p*<0.05, ***p*<0.01, ****p*<0.001

**Supplementary Table S1** The mRNA levels of Syncytin-1 in HCC and adjacent tissues were assessed by quantitative real-time PCR

| Tissue | + | - | Total | Positive rate (%) | *χ^2^* | *p* |
| --- | --- | --- | --- | --- | --- | --- |
| HCC | 28 | 5 | 33 | 84.85 | 26.826 | <0.001^***^ |
| NT | 7 | 26 | 33 | 21.21 |  |  |

^***^*p*<0.001

**Supplementary Table S2** The protein levels of Syncytin-1 in HCC and adjacent tissues were assessed by western blotting

| Tissue | + | - | Total | Positive rate (%) | *χ^2^* | *p* |
| --- | --- | --- | --- | --- | --- | --- |
| HCC | 27 | 6 | 33 | 81.82 | 21.959 | <0.001^***^ |
| NT | 8 | 25 | 33 | 24.24 |  |  |

^***^*p*<0.001

**Supplementary Table S3** Syncytin-1 expression in HCC and adjacent tissues were assessed by immunohistochemistry

| Tissue | + | - | Total | Positive rate (%) | *χ^2^* | *p* |
| --- | --- | --- | --- | --- | --- | --- |
| HCC | 84 | 19 | 103 | 81.55 | 48.389 | <0.001^***^ |
| NT | 13 | 40 | 53 | 24.53 |  |  |

^***^*p*<0.001

**Supplementary Table S4** Syncytin-1 promotes the formation of xenograft tumors in nude mice

|  | Overexpression of Syncytin-1 in NIH3T3 | | Downregulation of Syncytin-1 in HCCLM3 | |
| --- | --- | --- | --- | --- |
|  | pCMV | pCMV-Syncytin-1 | pSilencer-NC | pSilencer-shSyncytin-1 |
| No. of injected nude mice | 5 | 8 | 5 | 8 |
| No. of nude mice formed tumors | 0(0) | 7(87.5) | 5(100.0) | 1(12.5) |
| *p* | 0.005^**^ |  | 0.005^**^ |  |

^**^*P*<0.01

**Supplementary Table S5** Volumes (mm^3^) of xenograft tumors formed in nude mice

|  | Overexpression of Syncytin-1 in NIH3T3 | | Downregulation of Syncytin-1 in HCCLM3 | |
| --- | --- | --- | --- | --- |
| Day | pCMV | pCMV-Syncytin-1 | pSilencer-NC | pSilencer-shSyncytin-1 |
| Day 5 | 0 | 0 | 0 | 0 |
| Day 10 | 2.33±0.28 | 21.35±2.72^***^ | 14.25±1.57 | 2.15±0.26^***^ |
| Day 15 | 4.68±0.54 | 68.85±7.09^***^ | 51.06±5.23 | 5.22±0.57^***^ |
| Day 20 | 6.19±0.75 | 145.73±15.24^***^ | 124.32±12.06 | 6.34±0.68^***^ |
| Day 25 | 7.02±0.78 | 240.18±25.65^***^ | 210.61±22.48 | 6.65±0.71^***^ |
| Day 30 | 7.35±0.81 | 418.26±40.17^***^ | 390.74±40.14 | 7.18±0.72^***^ |

^***^*P*<0.001

**Supplementary Table S6** GO function analysis of upregulated genes associated with HCC

| GO:function category | count | *p* |
| --- | --- | --- |
| Gene to GO BP test for over-representation | | |
| GO:0007049~cell cycle | 103 | 0.000 |
| GO:0048285~ organelle fission | 61 | 0.000 |
| GO:0000280~nuclear division | 60 | 0.000 |
| GO:0051301~cell division | 60 | 0.000 |
| GO:0051726~regulation of cell cycle | 56 | 0.000 |
| GO:0051276~chromosome organization | 54 | 0.000 |
| GO:0007067~mitotic nuclear division | 53 | 0.000 |

**Supplementary Table S7** KEGG pathway analysis of upregulated genes associated with HCC

| Category of signalling pathway | ExpCount | count | *p* | Size |
| --- | --- | --- | --- | --- |
| Hsa04110: cell cycle | 2.065 | 19 | 0.000 | 122 |
| Hsa04114: Oocyte meiosis | 1.845 | 13 | 0.000 | 109 |
| Hsa05200: Pathway in cancer | 6.602 | 18 | 0.000 | 390 |
| Hsa04115: P53 signalling pathway | 1.515 | 7 | 0.000 | 68 |
| Hsa04010: MEK/ERK signalling pathway | 1.337 | 7 | 0.000 | 79 |
| Hsa05206: microRNAs in cancer | 2.539 | 9 | 0.001 | 150 |
| Hsa04068: Foxo signalling pathway | 2.218 | 7 | 0.007 | 131 |
| Hsa03460: Fanconi anemia pathway | 5.027 | 4 | 0.008 | 47 |
